# Changes in the faecal microbiome of pied tamarins (Saguinus bicolor) associated with chronic, recurrent diarrhoea and weight loss

**DOI:** 10.1186/s42523-020-00062-4

**Published:** 2021-01-05

**Authors:** Peter Richards-Rios, Paul Wigley, Javier López, Dominic Wormell, Alberto Barbón

**Affiliations:** 1 Institute of Veterinary Science, University of Liverpool, Liverpool, UK; 2 Institute of Infection and Global Health, University of Liverpool, Liverpool, UK; 3 Chester Zoo, Chester, UK; 4 Durrell Wildlife Conservation Trust, Channel Islands Jersey, France

**Keywords:** Microbiome, Pied tamarin, Wasting syndrome

## Abstract

**Background:**

Chronic recurrent diarrhoea and weight loss is a common problem in captive callitrichids. These symptoms are common clinical features of marmoset wasting syndrome (MWS), a chronic enteric inflammation of unknown aetiology associated with mortality in captive marmosets. The unknown aetiology of the condition presents problems for conservation projects where affected colonies present higher mortality and lower birth rates. Since a role for the microbiome has been established in chronic enteric inflammation of other species it is possible that the intestinal microbiome undergoes similar changes during MWS.

**Results:**

The faecal microbiome of pied tamarins (*Saguinus bicolor*) at Jersey Zoo was determined using 16S rRNA gene amplicon sequencing to compare the composition of the faecal microbiome of tamarins affected by chronic recurrent diarrhoea and weight loss with unaffected individuals. Affected individuals had a higher relative abundance of amplicon sequence variants assigned to *Lactobacillus* and *Helicobacter jaachi* while unaffected individuals had a higher relative abundance of some *Lachnospiraceae* and *Ruminococcaceae*.

**Conclusions:**

Although *Helicobacter* has been shown to reside in healthy wild and captive marmosets and tamarins and appears to form part of the normal microbiota, the results of this study raise the prospect that certain species of *Helicobacter* may be associated with chronic, recurrent diarrhoea in captive callitrichids. The presence of *Lactobacillus* may also play a role in the development of MWS. Since depletion of *Lachnospiraceae* and *Ruminococcaceae* have been linked to chronic gastrointestinal inflammation in humans, this feature of the microbiome of affected tamarins provides another avenue of further research in the pathogenesis of MWS.

**Supplementary Information:**

The online version contains supplementary material available at (doi:10.1186/s42523-020-00062-4).

## Introduction

The pied tamarin, *Saguinus bicolor*, is a species of callitrichid primate endemic to the Brazilian Amazonian rainforest, specifically close to the regional capital Manaus. This proximity to a large urban environment has resulted in habitat loss and fragmentation which threatens the wild population [26]. As a result, pied tamarins are classified by the IUCN as endangered [27]. Future survival may depend on successful captive breeding projects, however, pied tamarins have proved challenging to manage in captivity. One of the principal problems is their predisposition to develop chronic diarrhoea resulting in weight loss which appear to be symptoms of marmoset wasting syndrome (MWS), a chronic enteric disease of unknown aetiology associated with mortality in captive marmosets and other callitrichids. MWS is often described as one of the biggest problems facing captive callitrichid colonies [10, 29].

A variety of clinical signs of differing severities have been described as MWS. Most commonly MWS is defined as chronic diarrhoea accompanied by a reduction in body weight and cachexic changes such as muscle atrophy. Poor fur condition is often described which may progress to alopecia beginning at the tail base [46]. The condition has been associated with hypoalbuminaemia resulting from a protein-losing enteropathy [45, 46]. Gross lesions are most often reported in the colon, caecum and rectum [14]. Histologically, there is a chronic lymphoplasmacytic inflammation associated with focal necrosis and crypt obliteration progressing to ulceration [14]. Chronic colitis may progress to the development of neoplastic lesions [14]. The incidence of MWS in captive callitrichids has been documented, however, the poor clinical characterisation of the condition may lead to under- or overreporting [10]. A high mortality rate of 25 to 35% was reported among early attempts at maintaining colonies of captive callitrichids with adult mortality primarily caused by acute or chronic colitis and colonic adenocarcinoma [16, 17, 49]. Adenocarcinoma was not limited to older marmosets with cases reported as early as 15 months of age [17]. An improved understanding of callitrichid husbandry and better adaptation to captive conditions has lowered mortality among captive callitrichids although enteritis continues to be reported as a leading cause of death among adults [3]. Two other studies have identified colitis and colonic adenocarcinoma as a cause of death in between 10% to 23% of captive cotton-top tamarins [20, 35]. Considering that colonic adenocarcinoma has yet to be reported as a cause of mortality among wild cotton-top tamarins [61, 62], the high incidence in captivity can only be attributed to some factor associated with a captive state although MWS is not present in all captive colonies [29]. Some risk factors for the development of MWS have been suggested. Initially, a diet high in soft fruit and low in pelleted concentrate was associated with an increased risk of MWS [55]. Shimwell et al. [55] proposed that a high protein diet was required to prevent the development of MWS with the hypothesis further explored by other authors [9, 38]. However, a more recent study found that dietary crude protein was not a significant predictor for the development of MWS [10]. Instead, [10] suggest that low dietary fibre is a more important risk factor. Stress caused by proximity to predator species and lack of high plants around enclosures was also identified as a possible risk factor for MWS [10].

The clinical description of MWS and its associated risk factors of stress and low fibre diets show similarities to inflammatory bowel disease (IBD) in humans [6]. IBD encompasses a spectrum of morphological and clinical bowel disorders including Crohn’s disease and ulcerative colitis which are characterised by a chronic, often lymphocytic, inflammatory infiltrate of the lamina propria with variable changes to the overlying crypts and epithelium [41]. Due to these similarities, MWS has previously been used as an experimental model for IBD [16, 22] A component of IBD aetiology is the composition of the intestinal microbiota [28, 34]. Given other similarities between MWS and IBD it is reasonable to question whether the microbiota could play a role in MWS. However, the composition of the microbiota in callitrichids affected by MWS has not been investigated.

The intestinal microbiota of healthy captive marmosets has been studied using bacterial culture and compared to that of rhesus macaques [4]. Marmosets had significantly higher concentrations of aerobic and facultatively anaerobic Gram-negative bacteria and fewer anaerobic lactobacilli in the large intestine compared to rhesus macaques. Aerobic lactobacilli were not isolated from the small intestine of marmosets but were abundant in that of rhesus macaques [4]. One recent study aimed to compare the faecal microbiota of captive, translocated and wild marmosets. Wild marmosets had a higher alpha diversity compared to those in captivity. In terms of composition, captive marmosets had a higher relative abundance of Enterobacteriaceae while wild animals had a higher abundance of Helicobacteraceae, Bacteroidia, Bifidobacteriaceae and Veillonellaceae [42]. However, these samples were taken from healthy adults and any conclusion regarding the role of the microbiota in pathology would be speculative despite the presence of potentially pathogenic bacteria such as Enterobacteriaceae and Helicobacteraceae in both captive and wild samples. In terms of the effect of disease on the intestinal microbiota, one study found significant differences between the intestinal microbiota of healthy marmosets and those with chronic diarrhoea using T-RFLP analysis [54]. However, T-RFLP analysis doesn’t allow easy identification of taxonomic groups contributing to these differences. Additionally, quantitative PCR was used to measure differences in *Bifidobacterium* abundance between healthy and unhealthy animals showing that chronic diarrhoea was associated with lower levels of *Bifidobacterium* [54].

Currently, there is a lack of knowledge regarding the microbiota of healthy and diseased captive pied tamarins. Since the microbiome has been linked to a inflammatory bowel disease in humans, a role for the microbiome in chronic, recurrent diarrhoea in captive pied tamarins is also possible. Using animals from a single colony, this study aimed to investigate changes in the faecal microbiome of *S. bicolor* affected by chronic, recurrent diarrhoea and weight loss with unaffected animals that could help identify a potential cause or risk factor for this condition.

## Results

### Number of affected animals

Eight animals were classified as affected (5 males, 3 females, aged 12±6 years) and 13 as unaffected (10 males and 3 females, aged 6±3 years). 18 of 21 sampled animals had received oral antibiotics at some point in their lives prior to the health checks. The antibiotics used in affected animals included amoxicillin, amoxicillin/clavulanate, metronidazole, enrofloxacin, ciprofloxacin, erythromycin and trimethoprim/sulfamethoxazole. The number of different antibiotics used in each affected individual was 4±1. The antibiotics used in unaffected animals were amoxicillin/clavulanate, ciprofloxacin and trimethoprim/sulfamethoxazole. The number of different antibiotics used in each unaffected individual was 1±1. The number of days between the last day of treatment and sampling in the affected animals was 355±215 (min: 22, max: 1431) days and 869±1091 (min: 42, max: 3329) in unaffected animals. The total number of days that affected animals were treated with antibiotics during their lifetime was 618±735 (min: 21, max: 1824) while unaffected animals were treated for a total of 18±17 (min 5, max: 62) days.

### Sequencing effort

Insufficient DNA for 16S rRNA sequencing was extracted from two samples from affected tamarins. A total of 20 samples were included in 16S rRNA gene sequence analysis of which six were from affected tamarins, 13 were from unaffected tamarins and one community standard. A total of 3,720,753 reads were obtained from the samples submitted for sequencing. After filtering, merging of paired reads and chimera removal, a total of 2,818,464 reads remained (76% of the original total) giving a mean of 140,923 (±46,256) reads per sample. The median number of reads per sample was 146,090.

### Alpha diversity

**Fig. 1:**
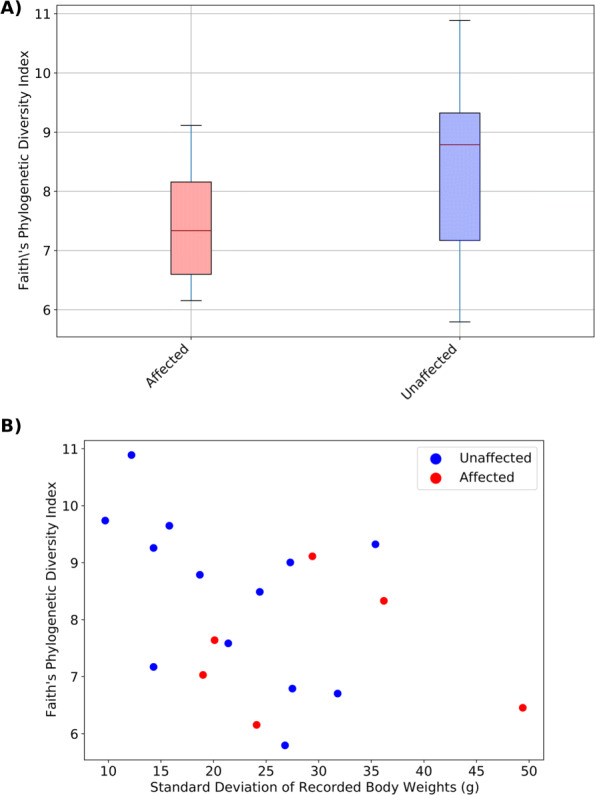
Alpha diversity. Affected animals had a higher species richness (**a**) than unaffected animals (Affected: mean = 7.45, SD = 1.13; Unaffected: mean = 8.40, SD = 1.47) although this difference was not significant (H = 1.97, *p* = 0.16). Species richness was negatively correlated with standard differentiation between recorded body weights (**b**) although the result was not significant (R = -0.44, *p* = 0.06)

The mean FPD index was lower in tamarins affected by diarrhoea, (Affected: mean = 7.45, SD = 1.13; Unaffected: mean = 8.40, SD = 1.47, Fig. 1a) although this difference was not significant (Test statistic = 1.97, *p* = 0.16). Since MWS is a chronic disease, often of an episodic nature, body weight can oscillate over time. Absolute body weights at sampling may not reflect this pattern. Instead, the standard deviation of body weights from previous health checks was used as a metric for variation in weight over time with a greater standard deviation expected in affected individuals. FPD index was negatively correlated with standard deviation between recorded body weights although the result was not significant (R = -0.44, *p* = 0.06, Fig. 1b). There were no correlations between FPD index and antibiotic administration or sex.

There was no significant difference in SD index between affected and unaffected tamarins (Test statistic = 0.49, *p* = 0.48). A negative correlation between standard deviation of recorded body weights and SD (R = -0.42, *p* = 0.07) was observed but was not significant.

### Beta diversity

**Fig. 2:**
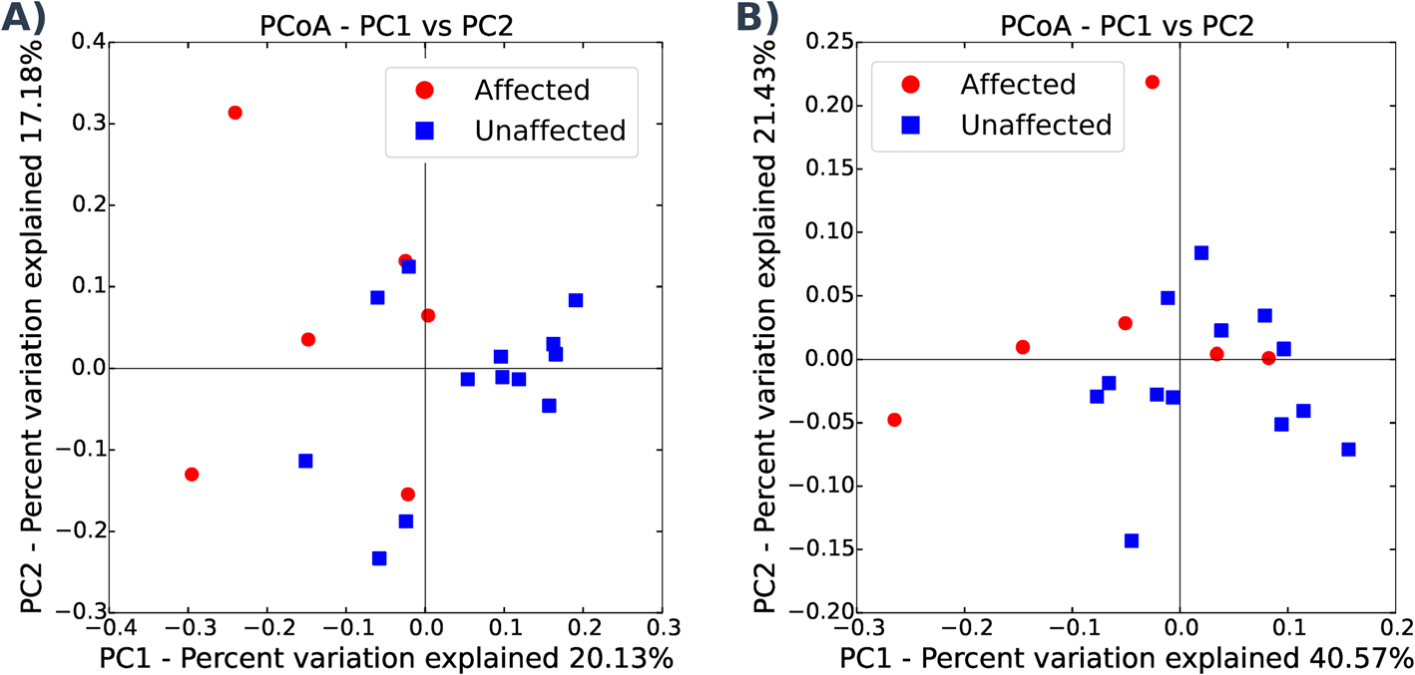
Unweighted and weighted beta diversity plots Disease status had a significant effect on unweighted (**a**) and weighted (**b**) beta diversity. The PCoA plots show some clustering of affected and unaffected samples although the low sample number makes interpretation of this clustering weak

Disease status had a significant effect on beta diversity measured using an unweighted UniFrac metric (ANOSIM statistic = 0.32, *p* = 0.027; PERMANOVA statistic = 2.13, *p* = 0.01). A PCoA plot of unweighted UniFrac distance between samples shows some clustering of affected and unaffected samples although the low sample number makes interpretation of this clustering weak (Fig. 2a). Although visually there appears to be more variation in beta diversity between affected samples compared to unaffected samples, a PERMDISP test revealed that there was no statistical difference in sample distance to the group’s spatial median between affected and unaffected groups (PERMDISP statistic = 1.76, *p* = 0.21).

Disease status continued to have a significant effect on beta diversity measured using a weighted UniFrac metric (ANOSIM statistic = 0.24, *p* = 0.04; PERMANOVA statistic = 2.42, *p* = 0.05). As with the unweighted UniFrac PCoA, although there is some clustering of samples according to disease status the low number of affected samples makes this clustering unconvincing (Fig. 2b). As for the unweighted UniFrac metric, a PERMDISP test did not detect a significant difference in samples distance to the group’s spatial median betweena ffected and unaffected groups (PERMDISP statistic = 3.25, *p* = 0.09).

### Differentially abundant ASVs

While visual assessment of taxa plots and subsequent statistical tests using relative abundances can be a useful initial aid in identifying differentially abundant taxa, these methods do not take into account the compositional nature of the data. Instead, Gneiss analysis was used to identify ASVs which were differentially abundant between affected and unaffected individuals. In order to reduce the number of ASVs in the analysis and remove low abundance ASVs, the feature table was filtered to exclude ASVs with a frequency of less than 21, reducing the number of ASVs included from 607 to 456. A dendrogram of principal balances was constructed containing 455 balances (y0-y454) which is displayed as part of Fig. 4. Isometric log ratios for each of these balances in each sample were calculated and compared between affected and unaffected individuals using a multivariate linear regression model with disease status as the only covariate. The overall linear regression model fit was R2 = 0.37 with covariate disease status accounting for 19.8% of variance.

**Fig. 3:**
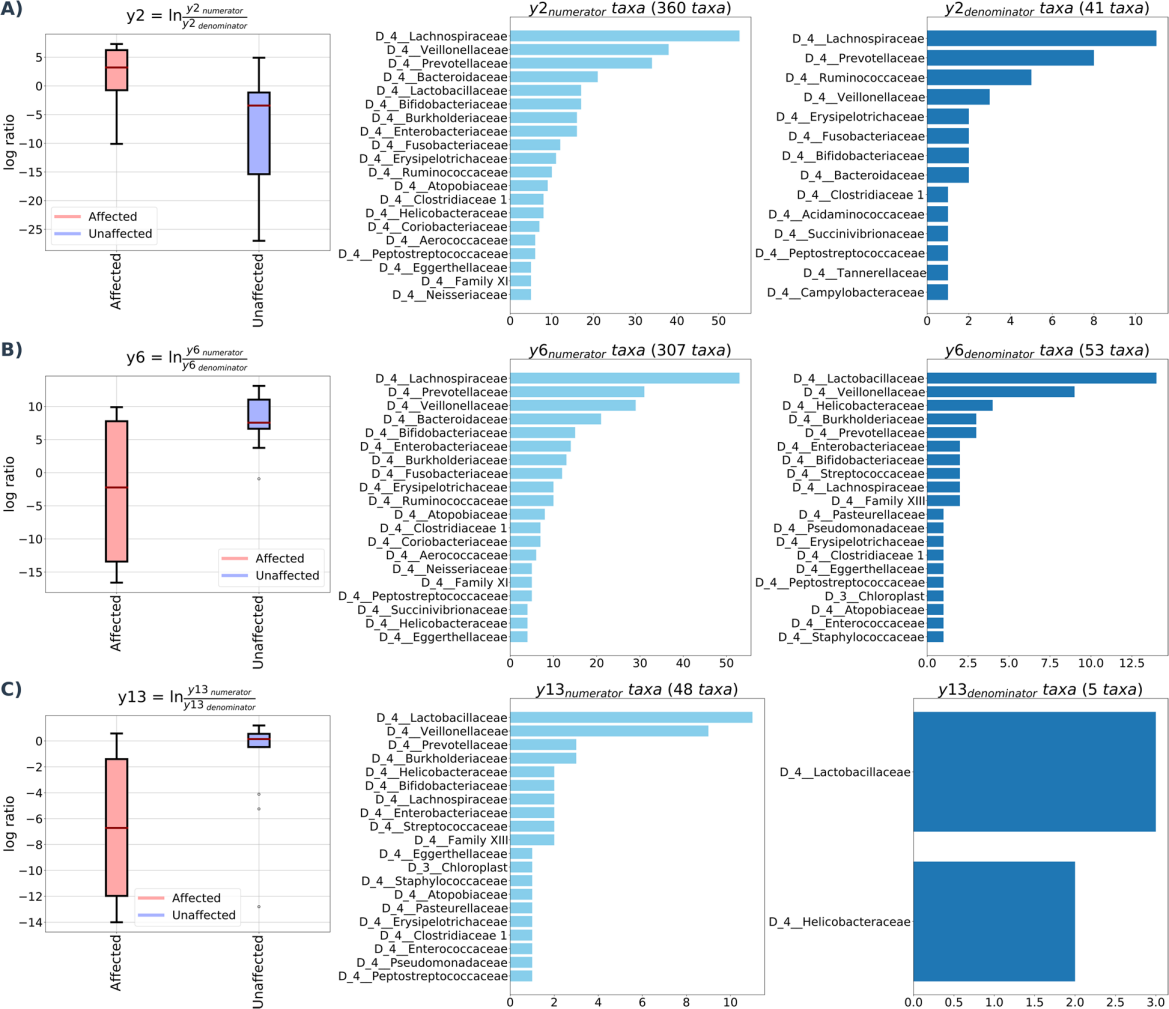
Log ratios of balances significantly different between affected and unaffected tamarins. The log ratios of three balances were significantly different between affected and unaffected tamarins: y2 (**a**), y6 (**b**) and y13 (**c**). A lower log ratio suggests a shift towards denominator taxa while a higher log ratio suggests a shift towards numerator taxa. The dendrogram heatmap (Fig. 4) and taxa plots (Fig. 5) can be used in conjunction with significant balances to identify taxa which are responsible for differences in log ratio between disease status

**Fig. 4:**
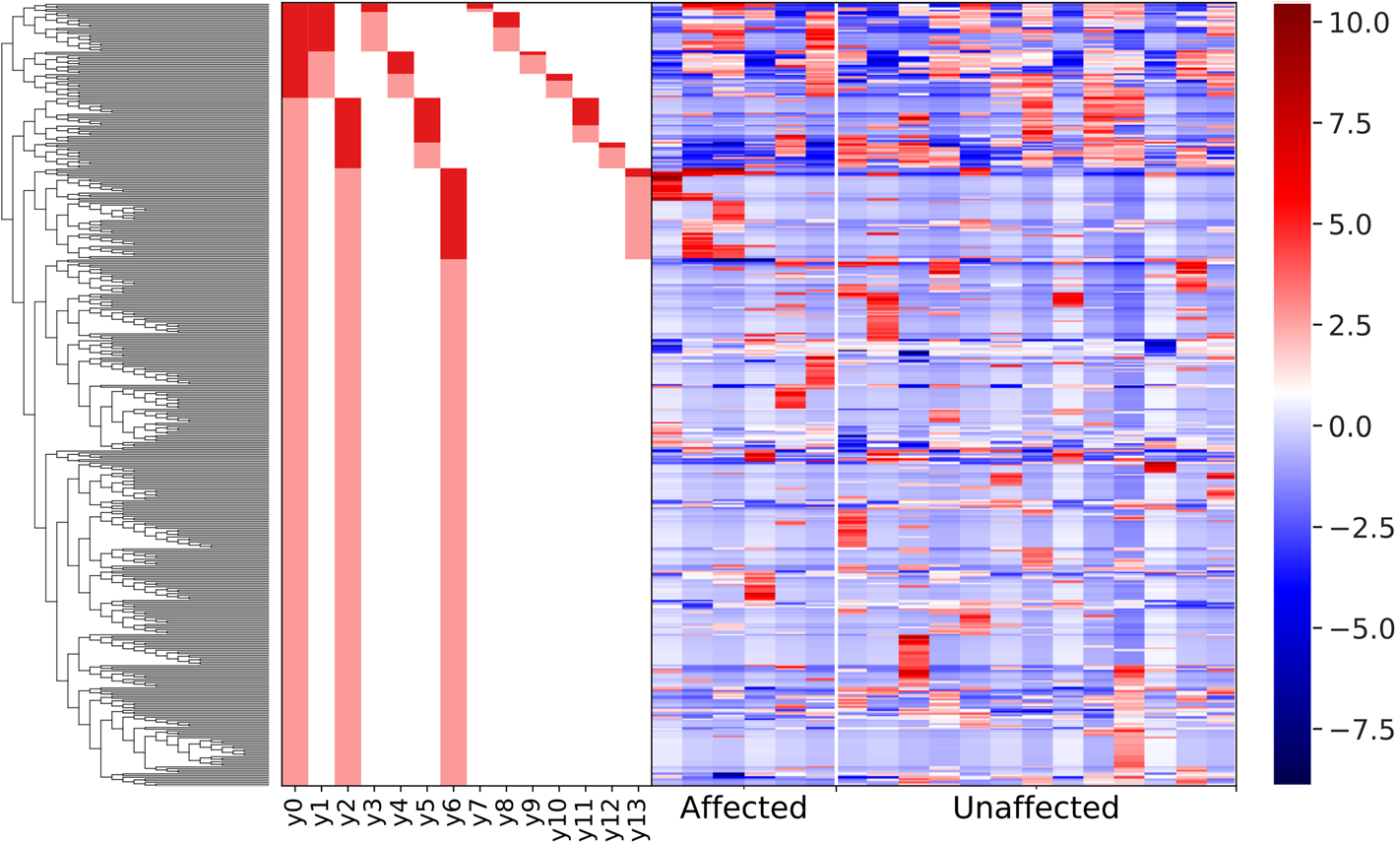
A dendrogram heatmap showing log abundance of features in the tamarin faecal microbiome. The heatmap allows resolution of significant balances with a higher abundance of y2 _denominator_ ASVs in unaffected tamarins while there was a higher abundance of y6 and y13 _denominator_ ASVs in affected tamarins

The linear regression model showed that balances y6 (β= 19.7, p <0.001) and y13 (β= 10.0, *p* = 0.002) were significantly affected by disease status. The dendrogram heatmap which displays the balance dendrogram, division of each balance into numerator and denominator ASVs and the log converted relative abundance of each ASV in individual samples arranged by disease status (Fig. 4) was reviewed. Balance y2 was considered to describe important differences between affected and unaffected tamarins as the relative abundance of some y2 _denominator_ ASVs appeared increased in unaffected compared to affected tamarins although there was not a significant different in the log ratio of balance y2 between groups (β= -14.1, *p* = 0.08). The log ratio of balances was displayed in box and whisker plots to visualise differences between groups. Log ratios and taxonomic composition of balances y2, y6 and y13 are available in Fig. 3. The log ratio of balance y6 was lower in affected tamarins compared to unaffected tamarins. The dendrogram heatmap (Fig. 4) shows that the relative abundance of y6 _denominator_ ASVs was higher in affected tamarins so explaining why the log ratio of this balance was lower in this group, however, the occurrence of these ASVs is sporadic with three samples showing high abundance while the remaining three samples showed low abundance.

Balance y13 is a subdivision of y6 _denominator_ ASVs (Fig. 4). The log ratio of balance y13 was significantly lower in affected tamarins (3). The dendrogram heatmap shows that y13 _denominator_ ASVs had a high relative abundance in four of six samples from affected tamarins compared to three of 13 samples from unaffected tamarins (Fig. 4). As for balance y6, this higher relative abundance of y13 _denominator_ ASVs is sufficient to explain the lower log ratio of this balance in affected tamarins. Overall, y6 _denominator_ ASVs, and by extension y13 _denominator_ ASVs were classified as having a higher abundance in affected tamarins.

The log ratio of balance y2 was higher in samples from affected tamarins compared to samples from unaffected tamarins although this difference was not significant (3). The dendrogram heatmap shows a higher relative abundance of y2 _denominator_ ASVs in unaffected tamarins (Fig. 4) explaining the lower log ratio in unaffected tamarins. As such these ASVs were classified as having a higher abundance in unaffected tamarins.

### Taxonomy of differentially abundant ASVs

**Fig. 5:**
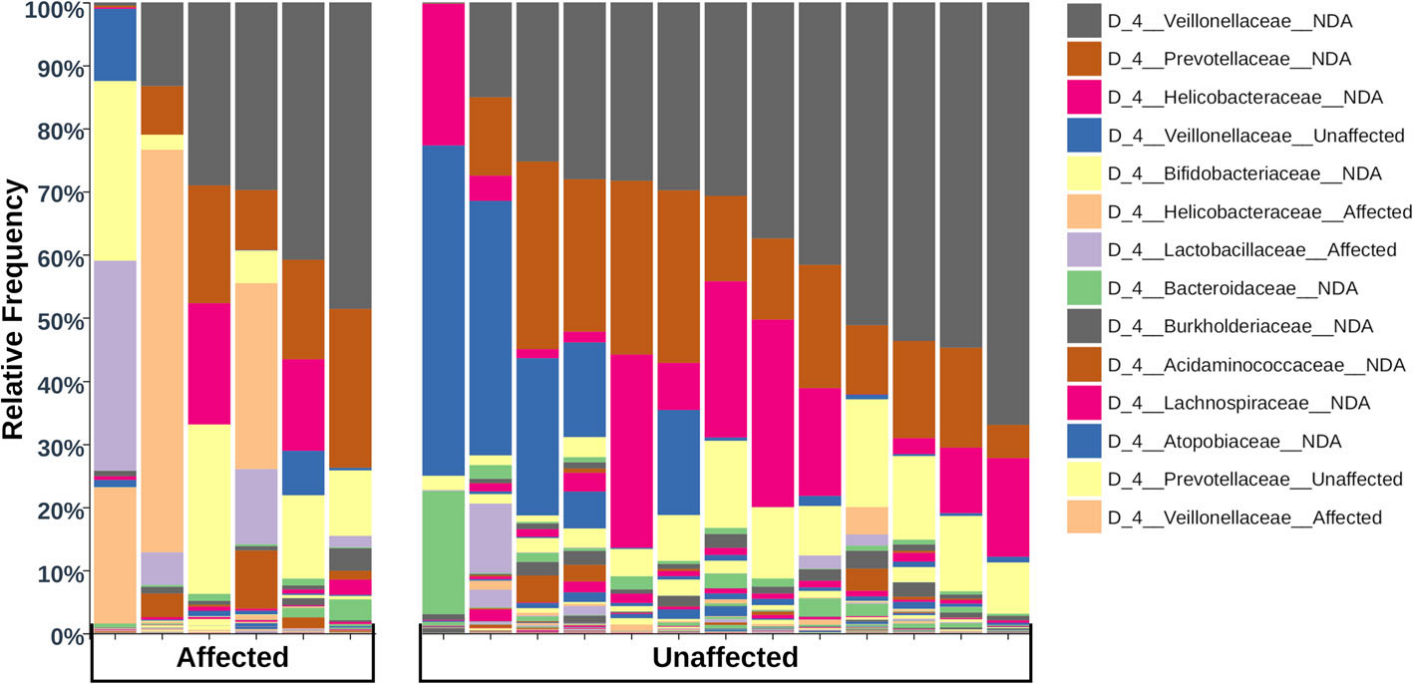
The relative abundance of bacterial families in the tamarin microbiome. The taxaplot also indicates whether contributing ASVs were classified as differentially abundant in unaffected (Unaffected) or affected (Affected) tamarins or not differentially abundant (NDA). ASVs assigned to *Lactobacillaceae* and *Helicobacteraceae* had a higher relative abundance in affected tamarins

Assessing differential abundance in taxonomy between sample groups can be challenging with many ASVs not assigned to species or even genera. For example, there may appear to be no difference in relative abundance of *Lactobacillus* when examined at the genus level when in fact the profile of ASVs assigned to *Lactobacillus* is different between groups. In order to overcome this difficulty, the taxa barplot (Fig. 5) shows the relative abundance of bacterial families in samples from affected and unaffected tamarins, however, each family is subdivided into three categories according to their classification by Gneiss analysis: not differentially abundant between groups (NDA), higher abundance in affected tamarins (Affected) and higher abundance in unaffected tamarins (Unaffected). The majority of ASVs were classified as NDA.

**Fig. 6:**
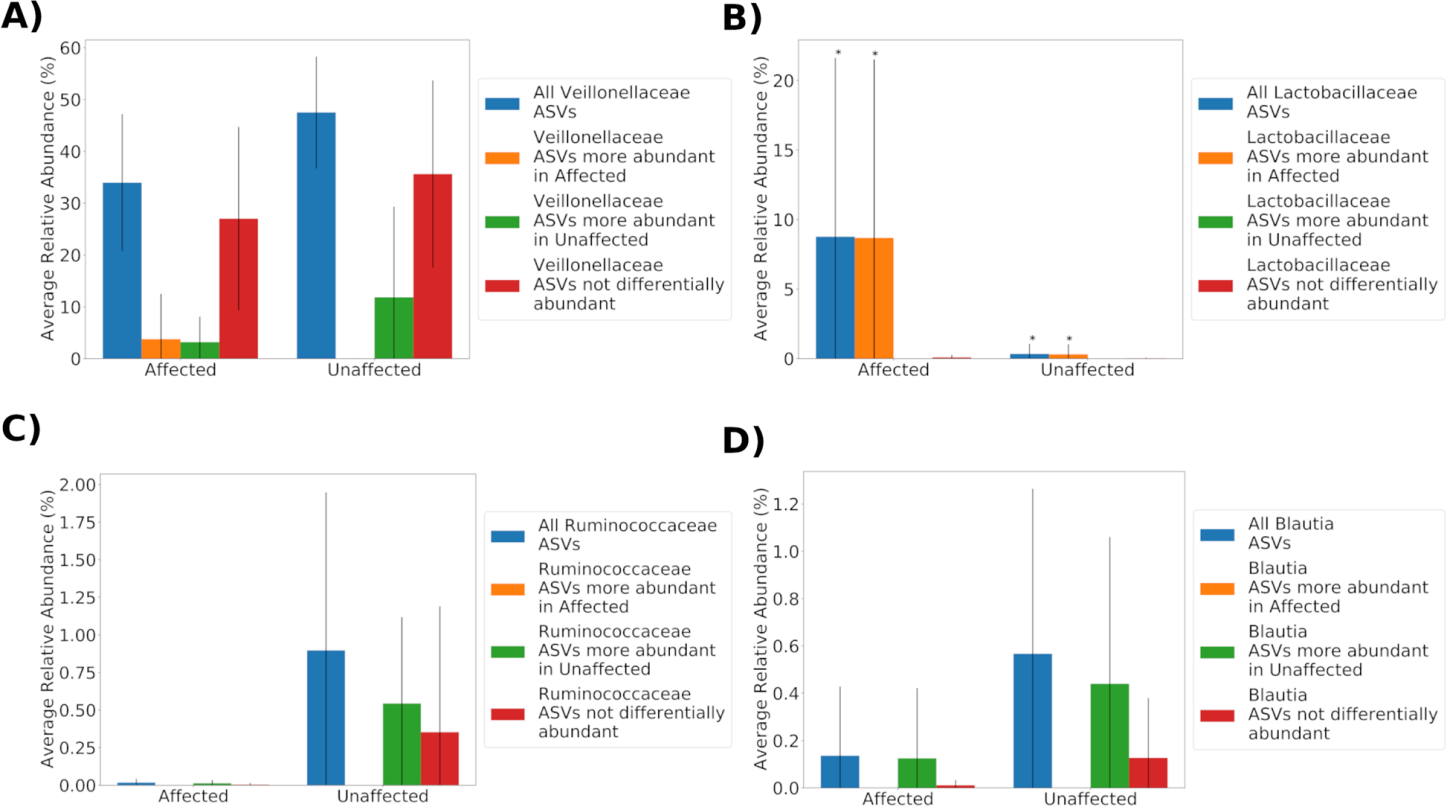
The average relative abundance of ASVs assigned to single bacterial taxa. The figures show relative abundance of ASVs assigned to bacterial taxa and grouped by classification as not differentially abundant (NDA), more abundant in affected tamarins and more abundant in unaffected tamarins. * indicates where the relative abundance of grouped ASVs was significantly different between unaffected and affected tamarins

The majority of relative abundance in both affected and unaffected samples is provided by ASVs that were not differentially abundant between groups and were assigned to Veillonellaceae, Prevotellaceae and Helicobacteraceae. Some Veillonellaceae and Prevotellaceae ASVs were classified by Gneiss as more abundant in unaffected samples, however, the chi-square test did not detect a significant difference in distribution of ASVs assigned to these taxa between the three Gneiss analysis classifications (NDA, Affected and Unaffected). Additionally, the relative abundance of Veillonellaceae was mainly composed of ASVs classified as NDA (Fig. 6a) in both affected and unaffected tamarins. Consequently, the classification of some Veillonellaceae ASVs as more abundant in affected or unaffected tamarins likely represents normal heterogeneity between individual intestinal microbiomes.

Table 1 shows the results of chi-square tests used to identify taxonomic groups at the family, genus and species level in which the distribution of ASVs between the three Gneiss classifications was significantly different to a random distribution. At the family level, significantly more ASVs assigned to Lactobacillaceae and Helicobacteriaceae were classified as more abundant in affected samples while significantly more ASVs assigned to Ruminocaccaeae were classified as more abundant in unaffected samples. In total 17 ASVs assigned to Lactobacillaceae were included in the analysis of which 14 were classified as more abundant in affected samples and three as NDA. Since it would be possible that the three Lactobacillaceae ASVs were contributing the majority of relative abundance in both affected and unaffected samples with negligible presence of other ASVs, the relative abundance of ASVs in each Gneiss classification were plotted with results shown in Fig. 6b. This shows that the majority of Lactobacillaceae relative abundance was contributed by ASVs identified as more abundant in affected samples where there was a significantly higher relative abundance of Lactobacillaceae. However, there was a large range of Lactobacillaceae relative abundance in affected samples with no Lactobacillaceae detected in two affected samples (Fig. 5).

**Table 1:** Taxonomic divisions containing ASVs identified as differentially abundant between affected and unaffected tamarins and were that significantly different to an expected distribution

Taxonomy	Number of ASVs				Chi-square test results	
	Total	Affected	Unaffected	NDA	Test statistic	Corrected *p*-value
**Family**						
D_4__*Lactobacillaceae*	17	14 (2)	0 (2)	3 (13)	82.9	<0.001
D_4__*Ruminococcaceae*	15	0 (2)	5 (1)	10 (12)	11.9	0.048
D_4__*Helicobacteraceae*	8	4 (1)	0 (1)	4 (6)	11.7	0.048
D_4__*Succinivibrionaceae*	1	0 (0)	1 (0)	0 (1)	10.1	0.063
**Genus**						
D_5__*Lactobacillus*	17	14 (2)	0 (2)	3 (13)	82.9	<0.001
D_5__*Blautia*	13	0 (2)	5 (1)	8 (10)	14.6	0.027
D_5__*Helicobacter*	8	4 (1)	0 (1)	4 (6)	11.7	0.063
D_5__*Lachnospiraceae* UCG-008	1	0 (0)	1 (0)	0 (1)	10.1	0.063
D_5__*Flavonifractor*	1	0 (0)	1 (0)	0 (1)	10.1	0.063
D_5__*Lachnospiraceae* NK3A20 group	1	0 (0)	1 (0)	0 (1)	10.1	0.063
D_5__*Sarcina*	1	0 (0)	1 (0)	0 (1)	10.1	0.063
D_5__*Roseburia*	1	0 (0)	1 (0)	0 (1)	10.1	0.060
**Species**						
D_6__*Helicobacter jaachi*	3	3 (0)	0 (0)	0 (2)	22.8	0.001
D_6__*Bacteroidaceae* bacterium DJF_B220	2	0 (0)	2 (0)	0 (2)	20.2	0.002
D_6__*Veillonella seminalis* ACS-216-V-Col6b	2	2 (0)	0 (0)	0 (2)	15.2	0.014
D_6__*Ruminococcus* sp. Marseille-P328	1	0 (0)	1 (0)	0 (1)	10.1	0.060
D_6__uncultured *Sarcina* sp.	1	0 (0)	1 (0)	0 (1)	10.1	0.060
D_6__*Lachnospiraceae* bacterium DJF_VP52	1	0 (0)	1 (0)	0 (1)	10.1	0.060
D_6__uncultured *Veillonellaceae* bacterium	1	0 (0)	1 (0)	0 (1)	10.1	0.060

Expected distributions are displayed in brackets

Eight ASVs assigned to Helicobacteraceae were identified in total with four classified as NDA and four as more abundant in affected samples. Helicobacteraceae ASVs classified as more abundant in affected samples were present in two affected samples and one unaffected sample, however, their relative abundance was higher in the affected samples (63.8% and 29.4% c.f. 4.3%). Given the importance of Helicobacteraceae in disease in other species, ASVs assigned to this family were examined at the species level. ASVs classified as NDA were assigned to *Helicobacter saguini* while those classified as more abundant in affected tamarins were assigned to *Helicobacter jaachi*. This is reflected in Table 1 where significantly more ASVs assigned to *Helicobacter jaachi* were more abundant in affected tamarins than would be expected. However, the sporadic occurrence of this species in affected samples significantly weakens any attempt to correlate its presence with disease.

Of the ASVs assigned to Ruminococcaceae, 10 were NDA and five were more abundant in unaffected samples. Figure 6c shows that Ruminococcaceae had a low relative abundance in affected samples. Unaffected samples had a higher total average abundance of Ruminoccaceae with ASVs classified as more abundant in affected samples contributing a large portion to total relative abundance. However, there was not a significant difference in Ruminococcaceae relative abundance between affected and unaffected tamarins. The most abundant genus of Ruminococcaceae was *Faecalibacterium* which was present in five unaffected samples at a higher relative abundance than affected samples where only one sample contained *Faecalibacterium*. However there was not a significant difference between the expected and actual number of *Faecalibacterium* ASVs classified as differentially abundant in affected or unaffected tamarins. One ASV assigned to *Flavonifractor*, a genus of Rumincoccaceae, was more abundant in unaffected tamarins.

Of the ASVs assigned to Lachnospiraceae, 58 were classified as NDA, 11 were more abundant in unaffected samples and two were more abundant in affected samples. The relative abundance of Lachnospiraceae ASVs classified as more abundant in affected samples was negligible. The most abundant genus of Lachnospiraceae was *Blautia*. Significantly more ASVs assigned to *Blautia* were classified as more abundant in unaffected tamarins than would be expected (Table 1). The relative abundance of *Blautia* was higher in unaffected samples (Fig. 6d). ASVs assigned to two other genera from Lachnospiraceae, Lachnospiraceae UCG-008 and *Roseburia*, were more abundant in unaffected samples, although only one ASV assigned to each was found in the study making any statistical associations weak.

LEfSe identified 27 discriminative features between groups, 24 of which were more abundant in unaffected and three of which were more abundant in affected tamarins. A summary plot of LEfSe results can be found as Supplementary Figure 1. Taxa identified as more abundant in unaffected samples included Veillonellaceae, in particular the genus *Megamonas*; members of Bacteroidales including members of Bacteroidaceae with Prevotellaceae; members of Clostridiales including *Anaerococcus*, *Lachnoclostridium* and genera in Ruminococcaceae (*Subdoligranulum* and *Flavonifractor*) and uncultured *Actinomyces*, *Collinsella* and *Proteus* species. Of the three discriminative features more abundant in affected tamarins, two were species of *Lactobacillus* and the other was a species of *Libanicoccus*. These results partially agree with those obtained via Gneiss. Species of Ruminococaceae and Lachnospiraceae were identified as higher abundance in unaffected tamarins while species of *Lactobacillus* were identified as higher abundance in affected tamarins by both methods. However, LEfSe did not identify significant differences in the abundance of *Helicobacter* species between the two groups. The identification of a higher abundance of *Lactobacillus* species in affected tamarins and members of Ruminococcaceae and Lachnospiraceae in unaffected tamarins by two methods slightly strengthens the argument that these taxa were differentially abundant between groups although this assessment continues to be hampered by the small sample size.

## Discussion

This study found significant differences between the microbiota of pied tamarins affected by chronic, recurrent diarrhoea and weight loss and unaffected individuals. Affected animals were also treated more frequently and for longer durations with a wider range of antibiotics. Given that antibiotic treatment has been shown to affect the microbiome of humans [30, 33], the cause of disturbances in the microbiota in affected marmosets may be related to higher levels of antibiotic treatment. However, results from the study must be interpreted in light of the small sample size used. The small sample size reflects the difficulties of gathering sufficient data from a critically endangered species which a small captive population. The limited numbers of tamarins used in the study reduces the statistical power of tests and overall reliability of significant results. With this caveat in place, some results from the study merit further discussion and could inform the direction of future research efforts.

ASVs assigned to Helicobacteraceae had a high relative abundance in most samples. This taxon was the second most abundant after Veillonellaceae and was detected in all but one sample. At the species level, most tamarins were colonised by *Helicobacter saguini* and ASVs assigned to this species were classified as NDA. However, four ASVs assigned to *Helicobacter jaachi* were classified as more abundant in affected tamarins, although the statistical basis for this classification was weak. Co-colonisation by *Helicobacter* species was not observed with either *H. jaachi* or *H. saguini* present in a sample. However, it could be argued that 16S rRNA gene sequencing of rectal swabs is not a sensitive test for the presence of pathogens within the gastrointestinal system with sensitivity further reduced by the use of low biomass samples. Based on the evidence from this study, a definite link between either *H. saguini* or *H. jaachi* cannot be made but their involvement in similar inflammatory enteric conditions in other species merits further investigation.

Both *H. saguini* and *H. jaachi* have been isolated from common marmoset faeces as well as a third species, *Helicobacter callitrichis* [52, 53, 60]. There is no clear association between the presence of *Helicobacter* and intestinal lesions in callitrichids. Species of *Helicobacter* are often reported to have been first isolated from callitrichids suffering from chronic colitis [49, 53] and have been shown to cause enteritis in a mouse model [53]. However, studies of the intestinal microbiota of healthy adult callitrichids, both wild and captive, often yield a high relative abundance of *Helicobacter* [1, 25, 42]. Furthermore, to date no studies have identified a positive correlation between inflammatory lesions and the presence of *Helicobacter* [18, 58]. However, our results found two species of *Helicobacter* within the microbiota of tamarins, with only one species associated with affected tamarins. The studies describing no relationship between the presence of *Helicobacter* and inflammatory lesions do not identify the species of *Helicobacter* present. The possibility that any association between *Helicobacter* and the presence of inflammation is species-specific should be further explored.

Equally, other factors may be influencing the expression of a pathogenic phenotype in *Helicobacter* just as the presence of *NetB*-toxin producing *Clostridium perfringens* is insufficient to cause necrotic enteritis in chickens without prior predisposing factors [32]. Lactobacillaceae were more abundant in samples from affected tamarins. In other species, Lactobacillaceae are normal commensals often associated with the small intestine [59]. However, previous studies of the marmoset microbiota have either not found Lactobacillaceae or found only low levels [4, 42]. *Lactobacillus* is able to ferment glucose to produce lactate and short chain fatty acids such as acetate [21]. These lower the pH of intestinal contents which is normally considered beneficial in terms of inhibiting the growth of potentially harmful bacteria such as Enterobacteriaceae. However, it could be questioned whether Lactobacillaceae are stable commensals in callitrichids given that studies of healthy adults have not found this usually common taxa in the intestinal microbiota. Due to their effect on the pH of intestinal contents, Lactobacillaceae could facilitate the binding of *Helicobacter* to intestinal mucin. A previous study showed that the ability of *Helicobacter pylori* to bind to human mucin is dependent on a low pH [37]. A similar association between environmental conditions and behaviour of *Helicobacter* isolated from callitrichids would be relatively easy to test in vitro.

As well as the presence of *Helicobacter* in nearly all tamarins, further differences of interest between affected and unaffected tamarins were observed. In particular, affected tamarins had a lower relative abundance of Lachnospiraceae with Ruminococcaceae largely absent in these samples. In contrast, unaffected samples had a higher relative abundance of Lachnospiracaeae and Ruminococcaceae. In particular, *Blautia* and *Faecalibacterium* were classified as more abundant in unaffected samples. These genera are of particular interest since they have previously been shown to have an anti-inflammatory effect in the gastrointestinal tracts of humans and other species [13, 15, 40, 48, 56]. Reduced levels of these taxa in affected tamarins is particularly interesting when considered in conjunction with risk factors for the development of MWS. Cabana et al. [10] suggest that dietary fibre may have a protective effect against MWS. Studies in other species have highlighted the beneficial effects of high dietary fibre on gut health in relation to altering the composition of the microbiome, including increased abundance of *Faecalibacterium* [5, 36]. Furthermore, a study following the change in faecal microbome associated with seasonal dietary changes in wild black howler monkeys (*Alouatta pigra*) found that increased dietary fibre was linked to an increase in the relative abundance of Ruminococcaceae [2]. As well as dietary fibre, [10] highlighted the importance of stress as a risk factor for MWS. The effect that stress has on the microbiome is less well characterised although stressful events have been linked to the worsening of symptoms in humans with IBD [6]. Consequently, since low levels of *Faecalibacterium* have been linked to IBD in humans, the possiblity that stress negatively impacts the relative abundance of *Faecalibacterium* in the gut has been suggested [24]. In light of results from studies showing no correlation between *Helicobacter* and inflammatory disease in marmosets, it is possible that a more complex pathogenesis is required for development of clinical disease. While the presence *Helicobacter* alone is not sufficient to cause disease, depletion of beneficial microbiota either through stress or insufficient dietary fibre could allow an opportunity for *Helicobacter* to establish infection.

## Conclusion

The results of this study have demonstrated some differences in intestinal microbiome between pied tamarins exhibiting symptoms of MWS and unaffected individuals. The principal differences were a higher abundance of ASVs assigned to *Helicobacter jaachi* and *Lactobacillus* in affected tamarins while the relative abundance of ASVs assigned to *Lachnospiraceae* and *Ruminococcaceae* were higher in unaffected tamarins. Although a link between *Helicobacter* and MWS has been postulated, previous studies have not found a difference in *Helicobacter* abundance between animals affected and unaffected by MWS. However, since these studies have not been conducted at the species level, it is possible that *Helicobacter saguini* and *Helicobacter jaachi* behave differently within the gastrointestinal tract. Additionally, there is a possibility that modulation of the gastrointestinal environment by *Lactobacillus* could enhance the pathogenicity of *Helicobacter*. The lower abundance of *Lachnospiraceae* and *Ruminococcaceae* in affected tamarins provides another avenue of exploration in terms of pathogenesis of MWS. Depletion of these taxonomic groups have been linked to chronic gastrointestinal inflammation in humans, and a similar process could be at work in animals affected by MWS. Further research is required to explore the influence of *Helicobacter*, *Lactobacillus*, *Lachnospiraceae* and *Ruminococcaceae* on the development of MWS in tamarins and marmosets.

## Methods

### Pied tamarin colony

Pied tamarins housed at Jersey Zoo were used in this study. Tamarins all had permanent access to large indoor and outdoor areas, predominantly in buildings housing 3-5 callitrichid groups [63]. Indoor cages were of broadly similar size; minimum dimensions were approximately 2.25 m high x 1.53 m wide x 2.45 m deep. All indoor areas received natural light via skylights or windows. In addition, artificial lighting was provided via strip lights and heat lamps from 0800 to 1800. In the winter months, supplementary UV lighting was also put in place [39]. Outside enclosures were 16–63 m2 in area and approximately 4 m high, and were planted with extensive natural vegetation as well as being furnished with ropes, branches and platforms. The design of the buildings meant that tamarins had no visual contact while in their indoor areas, and no or very limited visual contact outside. Levels of auditory and olfactory contact were similar in all buildings. Three buildings were on show to the public, but only the outdoor areas were accessible to visitors, and there were standoff barriers averaging 1 m from the front of each enclosure, reducing the opportunity for visitors to touch the animals or the mesh cage fronts. Three other buildings were off-show.

The diet offered to the pied tamarins has changed since the establishment of the colony. At the time of this study the animals were offered three meals per day with one meal provided in the morning (08:00-08:30), at midday (12:00-13:00) and in the afternoon (15:30-17:00). The morning meal was a mixture of a commercial pellet diet (Mazuri New World Primate Biscuit, Mazuri Exotic Animal Nutrition, St. Louis, 63166, United States) soaked in warm water with honey and a banana puree prepared using bananas, arabic gum, aloe vera, sunflower or olive oil and calcium lactate. Vitamin D is added during the winter months. At midday the animals were offered a mixture of fruit and vegetables; carrot, plum, pear, cucumber, sweet potato, potato, papaya, avocado, bananas and occasionally nectarines and pomegranates. During this meal animals were also given peanuts, eggs or commercial cat food (Whiskas cat food chicken in jelly, Mars UK, Slough SL14JX, United Kingdom). The items are rotated through the different days of the week. In the evening locusts and waxmoth larvae are offered. Tamarin enclosures were cleaned in the morning, and excess food removed in the late afternoon. Tamarins were trained to sit on scales within their enclosures and were weighed at least weekly.

Recurrent weight loss and chronic diarrhoea have been observed in the colony over the years. Ante mortem and post mortem findings resemble those described in other callitrichid species as wasting syndrome or marmoset wasting syndrome [10, 14, 50, 57] but the disease has not been characterized in pied tamarins.

### Selection of animals and sampling

Thirty seven pied tamarins were housed at Jersey zoo between November 2018 and April 2019. During this period 21 animals, (15 males and 6 females) aged between 2 and 21 years, underwent routine health checks. Animals were anaesthetised using isoflurane and oxygen, a health check comprising a physical examination, blood sampling and radiographs was carried out. Blood samples were submitted for routine hematology and biochemistry. At the time of sampling, no animals were suffering from diarrhoea. A rectal swab was collected by introducing a fine tip cotton swab 10cm into the rectum. Once removed from the rectum, the tip of the swab was cut using sterile scissors and placed in a tube containing a nucleic acid and bacterial inactivation preservative (DNA/RNA Shield, Cambridge Bioscience, Cambridge, CB238SQ, United Kingdom). Samples were stored at room temperature (15−20^∘^C) until DNA extraction.

**Fig. 7:**
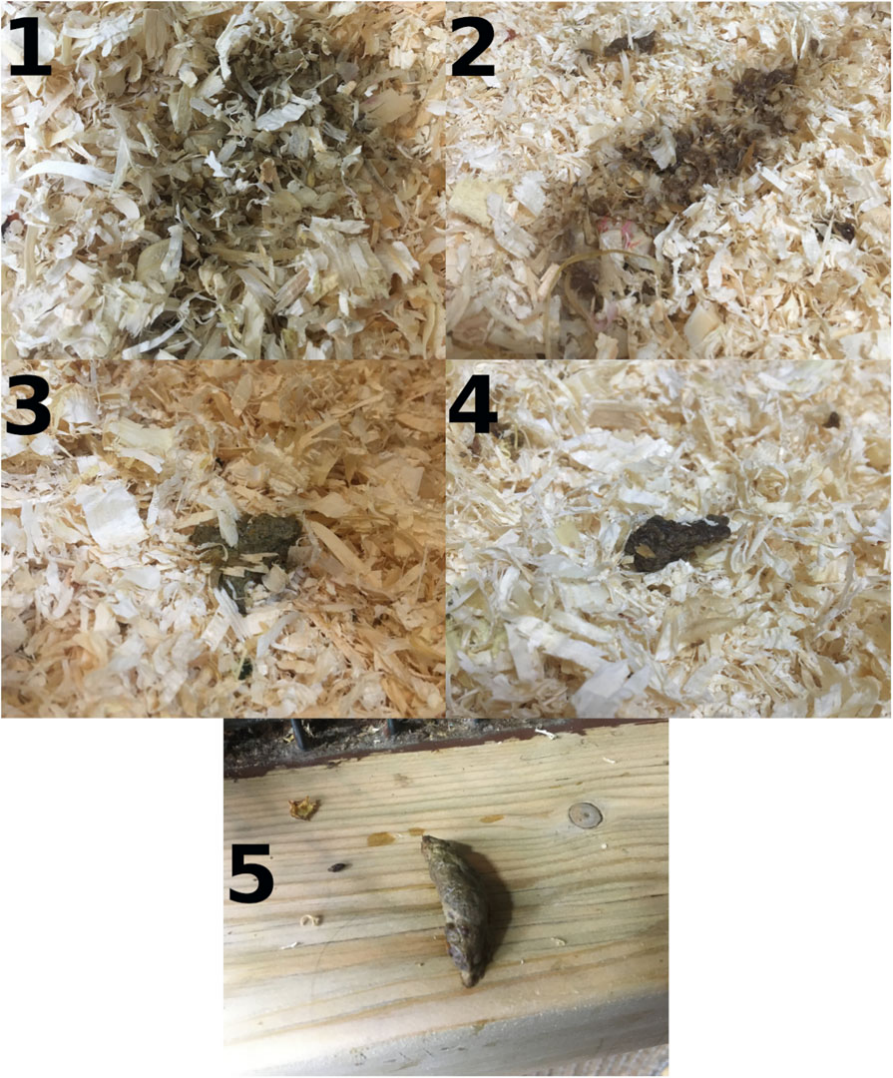
Faecal scoring guide in pied tamarins. Grade 1: very liquid faeces, no evidence of large particles, spread over large area; Grade 2: Very soft faeces, spread over small area, some solid particles present; Grade 3: Faeces have pasty consistency, no significant spread, not forming a pellet; Grade 4: Faeces forming a pellet but fragmented and showing soft consistency.; Grade 5: Faeces forming a pellet that maintains shape and integrity when manipulated

Medical and husbandry records from the animals were reviewed to establish if they had recurrent episodes of diarrhoea accompanied by body weight loss consistent with marmoset wasting syndrome. This data was used to classify animals as either affected or unaffected. Animals were defined as affected if they had undergone 3 or more episodes of diarrhoea during their lifetime which lasted for 5 or more days and during which they lost more than 10% of their body weight. Presence of diarrhoea was determined by subjective assessment of the faecal consistency and appearance by animal keepers using the scale shown in Fig. 7. A faecal grade equal or less than 2 was classified as diarrhoea.

Oral antibiotic prescriptions were reviewed in all the animals. The specific antibiotic used was recorded as well as, the number of days between sampling and the last day of antibiotic administration. The total number of days which animals received oral antibiotics during their lifetime was recorded.

### DNA extraction

DNA was extracted from each sample using Zymobiomics DNA MicroKits (Cambridge Bioscience, UK) according to the manufacturer’s instructions. DNA was extracted from swabs with an initial bead-beating step performed using a Qiagen TissueLyser at 30Hz for 10 minutes. DNA was extracted from all samples at the same time. At extraction, two controls were included: a blank extraction to control for contamination and 75µl of Zymobiomics Standard Bacterial Community (Cambridge Bioscience, UK) to control for variations in DNA extraction efficacy. Extracted DNA was quantified using a NanoDrop 2000 spectrophotometer (NanoDrop Technologies) and a Qubit dsDNA HS fluorometric kit (Invitrogen).

### Illumina MiSeq sequencing

Extracted DNA was sent for paired-end sequencing of the 16S rRNA gene at the Centre for Genomic Research (University of Liverpool) using an Illumina MiSeq run. The V4 hypervariable region (515F/R806) was amplified to yield an amplicon of 254 base pairs [12]. Library preparation was performed using a universal tailed tag design with subsequent amplification performed using a two step PCR with a HiFi Hot Start polymerase (Kapa) [19]. The first round of PCR was performed using the primers 5’-ACACTCTTTCCCTACACGACGCTCTTCCGATCTNNNNNGTGCCAGCMGCCGCGGTAA-3’ (forward) and 5’-GTGACTGGAGTTCAGACGTGTGCTCTTCCGATCTGGACTACHVGGGTWTCTAAT-3’ [19]. The raw Fastq files were trimmed for the presence of Illumina adapter sequences using Cutadapt version 1.2.1. The reads were further trimmed using Sickle version 1.200 with a minimum window quality score of 20. Reads shorter than 10 base pairs after trimming were removed.

### Amplicon sequence variant identification and taxonomy assignment

QIIME2 version 2018.4.0 was used for analysis of the Illumina data [8]. Amplicon sequence variant (ASV) assignment was completed using the dada2 plugin [11] and a feature table produced using the feature-table plugin [43]. Taxonomy was assigned using a pre-trained NaiveBayes classifier based on the SILVA 132 database of the 515F/R806 region of the 16S rRNA gene [64], available for download at https://docs.qiime2.org/2018.11/data-resources/, using the q2-feature-classifier plugin [7].

### Data analysis and statistics

Alpha and beta diversity analyses were performed at a sampling depth of 26,000 using the alignment [31], phylogeny [47] and diversity (https://github.com/qiime2/q2-diversity) plugins. Alpha diversity was measured using Faith’s phylogenetic diversity (FPD) index [23] to assess species richness and a Shannon diversity (SD) index to assess species evenness. Alpha diversity was compared between sample groups using a Kruskal Wallis test with a false discovery rate (FDR) correction for categorical data and a Spearman Rank correlation for numerical data. Taxa plots were produced using the q2-taxa plugin (https://github.com/qiime2/q2-taxa). Beta diversity, a metric used to compare species diversity and abundance between samples, was calculated with weighted and unweighted UniFrac metrics. The beta diversity matrix was used to draw principal coordinate analysis (PCoA) plots and ANOSIM and PERMANOVA tests over 999 permutations were used to determine the significance of differences in beta diversity between groups. Subsequently, a PERMDISP test was used to assess variance in distance between the sample group’s spatial median.

Gneiss analysis was chosen to analyse differential abundance of ASVs between groups since it overcomes challenges created by the compositional nature of microbiota data. Firstly, a dendrogram of ASVs is prepared using correlation clustering. Each node in the dendrogram is treated as a ’balance’ with taxa on one side of the balance termed numerators and on the other, denominators. Gneiss analysis examines the log ratio of abundances between numerator and denominator taxa at each balance. Each log ratio’s final numerical value is dependent on the balance between the taxa composing the numerator and those composing the denominator of the ratio. Differences in the log ratio of a balance can be compared between sample groups to determine differences in microbiota composition. A significant difference between samples allows hypotheses to be formulated regarding changes in the absolute abundance of numerator and denominator taxa but gives no further information as to which hypothesis is correct. For example, if balance y0 is found to be significantly lower at Group A compared to Group B the following hypotheses could explain the result: i) The numerator taxa are increased between Groups A and B; ii) The denominator taxa are decreased between Groups A and B; iii) A combination of hypotheses i) and ii); iv) Both numerator and denominator taxa are increased between Groups A and B, but numerator taxa are increased more; v) Both numerator and denominator taxa are decreased between Groups A and B, but denominator taxa are decreased more. Further investigations, such as quantitative PCR, are required to discern which hypothesis is correct [44], however in the case of this experiment, the volume of extracted DNA was not sufficient for further investigations.

Gneiss analysis [44] was run using QIIME2 to identify taxa which were differentially abundant between time points and area sampled. First, the ASV table was filtered to exclude low abundance ASVs. The count threshold for exclusion of ASVs was set at the first quartile to exclude the lowest 25% of ASVs by total frequency across all samples. Principal balances for use in Gneiss were obtained via Ward’s hierarchical clustering using the correlation-clustering command producing a dendrogram. Isometric log ratios for each balance were calculated using the ilr-transform command. A multivariate response linear regression model of log ratios balances was constructed with disease status as the only covariate using the ols-regression command. Results were visualised through a regression summary and dendrogram heatmaps. Balances significantly affected by the disease status were identified as those with a *p*-value less than 0.05.

The taxonomy of ASVs identified by Gneiss analysis as more abundant in affected and unaffected tamarins or not differentially abundant (NDA) was examined at the level of family, genus and species. A chi-square test was used to assess the distribution of taxa across Gneiss analysis groups. The proportion of total ASVs classified to each group (affected, unaffected, NDA) was calculated. This proportion was applied to the total number of ASVs assigned to each taxon to generate an expected number of each taxon in each group. P values were corrected for multiple tests using an FDR correction.

In order to strengthen evidence for identification of taxa which were differentially abundant between affected and unaffected tamarins, a second method was used to corroborate results from Gneiss. The taxonomy of the ASV table used as Gneiss input was collapsed to species level. This collapsed table was used as input for LEfSe (Linear discriminant analysis effect size) to identify bacterial taxa whose relative abundance was significantly different between affected and unaffected tamarins [51].

## Supplementary Information

**Additional file 1** Supplementary Figure 1.

## Data Availability

The datasets generated for this study can be found in the NCBI Sequence Repository Archive under bioproject number PRJNA609889.

## Abbreviations

ASV: Amplicon sequence variant
DNA: deoxyribonucleic acid
FPD: Faith’s phylogenetic diversity
IBD: Inflammatory bowel disease
IHC: Immunohistochemistry
IUCN: International Union for Conservation of Nature
MWS: Marmoset wasting syndrome
NDA: Not differentially abundant
PCoA: Prinicipal coordinate analysis
PCR: Polymerase chain reaction
rRNA: ribsomal ribonuclei acid
T-RFLP: Terminal restriction fragment length polymorphism
SD: Shannon diversity
