# Supplementary material for: Changes in the faecal microbiome of pied tamarins (Saguinus bicolor) associated with chronic, recurrent diarrhoea and weight loss
Source: Anim Microbiome. 2021 Jan 5;3:1. doi: 10.1186/s42523-020-00062-4 (PMC7934480; doi:10.1186/s42523-020-00062-4)
Supplement: Supplementary file 1 — Additional file 1 Supplementary Figure 1. [file 42523_2020_62_MOESM1_ESM.docx]

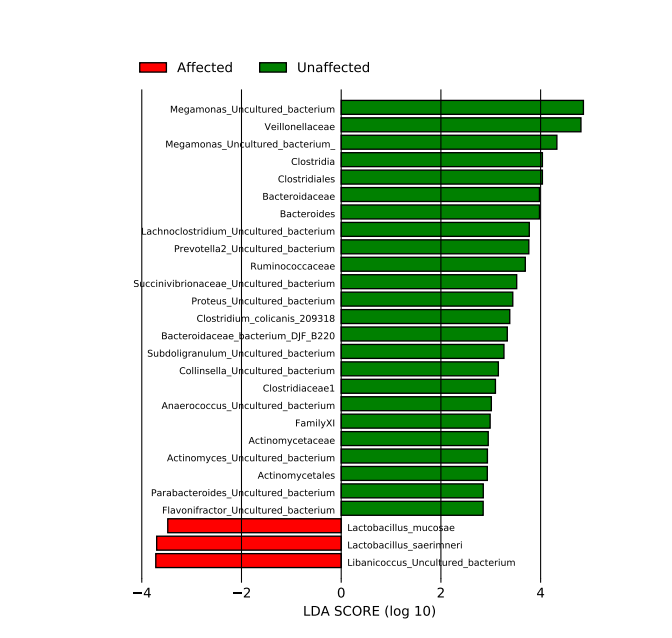
**Supplementary Figure 1 -** Plot showing the linear discriminant analysis (LDA) score for taxa which were significantly more abundant in Affected and Unaffected tamarins as determined by LEfSe
